# Osmolytes dynamically regulate mutant Huntingtin aggregation and CREB function in Huntington’s disease cell models

**DOI:** 10.1038/s41598-020-72613-3

**Published:** 2020-09-23

**Authors:** Shreyaas Aravindan, Samantha Chen, Hannaan Choudhry, Celine Molfetta, Kuang Yu Chen, Alice Y. C. Liu

**Affiliations:** 1 Department of Cell Biology and Neuroscience, Rutgers State University of New Jersey, Nelson Biology Laboratory, 604 Allison Road, Piscataway, NJ 08854 USA; 2 Department of Chemistry and Chemical Biology, Rutgers State University of New Jersey, Nelson Biology Laboratory, 604 Allison Road, Piscataway, NJ 08854 USA

**Keywords:** Biochemistry, Neuroscience, Diseases

## Abstract

Osmolytes are organic solutes that change the protein folding landscape shifting the equilibrium towards the folded state. Herein, we use osmolytes to probe the structuring and aggregation of the intrinsically disordered mutant Huntingtin (mHtt) vis-a-vis the pathogenicity of mHtt on transcription factor function and cell survival. Using an inducible PC12 cell model of Huntington’s disease (HD), we show that stabilizing polyol osmolytes drive the aggregation of Htt103Q^Exon1^-EGFP from a diffuse ensemble into inclusion bodies (IBs), whereas the destabilizing osmolyte urea does not. This effect of stabilizing osmolytes is innate, generic, countered by urea, and unaffected by HSP70 and HSC70 knockdown. A qualitatively similar result of osmolyte-induced mHtt IB formation is observed in a conditionally immortalized striatal neuron model of HD, and IB formation correlates with improved survival under stress. Increased expression of diffuse mHtt sequesters the CREB transcription factor to repress CREB-reporter gene activity. This repression is mitigated either by stabilizing osmolytes, which deplete diffuse mHtt or by urea, which negates protein–protein interaction. Our results show that stabilizing polyol osmolytes promote mHtt aggregation, alleviate CREB dysfunction, and promote survival under stress to support the hypothesis that lower molecular weight entities of disease protein are relevant pathogenic species in neurodegeneration.

## Introduction

A distinct and common observation in age-related neurodegenerative disease (ND) is that disease proteins aggregate forming insoluble protein deposits and inclusion bodies^1–4^. Examples include neuritic plaques and neurofibrillary tangles in Alzheimer’s disease, Lewy bodies in Parkinson’s disease, and inclusion bodies (IB) in Huntington’s disease. A long held prevailing hypothesis is that abnormal protein deposition triggers a complex neurotoxic cascade to manifest over time in neurodegenerative disease (ND). Accordingly, research and therapeutics development efforts have largely focused on the prevention and removal of disease protein aggregates, an approach with limited progress thus far^5^. This background along with the noted discordance of disease protein deposition vis-a-vis the pathology of neuron vulnerability and death, raise the possibility that while the insoluble end-stage species of protein aggregation are hallmarks of ND, they may not be the primary pathogenic agents^6–8^.

Multiple studies suggest that lower molecular weight or precursor forms of disease protein aggregate—including monomers, oligomers, proto-fibrillar, and non-fibrillar protein assemblies—may be the perpetrators of neurotoxicity in ND^6–8^. Our work aims to understand the cellular dynamics that govern the structuring and aggregation of the polyQ-expanded mutant Huntingtin protein (mHtt) in relation to the pathogenic process in Huntington’s disease. In a previous study, we reported that the induction of HSP chaperones by heat shock promotes the compaction of diffuse mHtt into IBs. This is coincident with the de-repression of cAMP-response element binding protein (CREB), heat shock factor 1 (HSF1), and nuclear factor κ light chain enhancer of activated B cells (NFκB) function, transcription factors implicated in memory formation, stress resistance and neuron survival^9^. This work adds to a growing stream of evidence that diffusible entities of disease proteins can drive pathogenesis, in part by binding to and quenching key regulatory proteins and the aggregation of disease proteins may represent a coping mechanism or consequence in disease states^2,6,7,10^.

The effects of transient heat shock in driving mHtt IB formation is dependent on the production of HSP chaperones^9^, proteins widely acknowledged to play important roles in protein folding, structuring, and quality control^11,12^. Here, we used osmolytes as tools to further assess the role of protein structuring in driving the assembly and aggregation of the intrinsically disordered polyQ-Htt^exon1^ protein into IBs. Osmolytes are naturally occurring, small organic molecules that accumulate in cells for protection against denaturing stresses^13–17^. The chemical classes of osmolytes include polyol/carbohydrates, amino acids, and methylamines. Functionally, osmolytes can be categorized into (i) stabilizing osmolytes (kosmotropes) including glycerol, sorbitol, sucrose, and trehalose used in this study, and (ii) destabilizing osmolytes (chaotrope) with urea being a prime example. The manner by which stabilizing osmolytes bolster protein structure and function is attributable to their direct effects on protein surface and peptide backbone, and indirect effect on water structure^15,16^. This solvophobic or osmophobic effect raises the chemical potential of the unfolded/unstructured state of proteins, thus shifting the folding equilibrium towards more compact structures with lower solvent accessible surface area^15,16^. The effect of osmolytes on protein structuring is biologically compatible, and ubiquitous. The term “chemical chaperone” underscores the innate and generic activity of osmolytes in promoting protein folding^18^. In fact, studies show that osmolytes can rescue mutant proteins by re-directing their folding and routing for proper function^19^.

In this work, we report that physiologically relevant concentrations of stabilizing polyol osmolytes share the generic activity of promoting the aggregation of intrinsically disordered mHtt from a diffusible ensemble into IB with beneficial outcomes in CREB function and cell survival.

## Results

### Osmolytes drive the assembly and aggregation of diffuse mHtt into forming inclusion bodies

We use the 14A2.6 line of PC12 cells with stably integrated ecdysone receptor based inducible expression of the Htt103Q^Exon1^–EGFP chimera as a tractable cell model of Huntington’s disease^20^. Our previous study showed that ponasterone A (PA, 5 µM) robustly induces the expression of the normal Htt (25Q) and the polyQ expanded mHtt (103Q) proteins in their respective cell lines, but only the mHtt protein forms inclusion bodies (IBs) under appropriate experiment conditions^9^.

**Figure 1:**
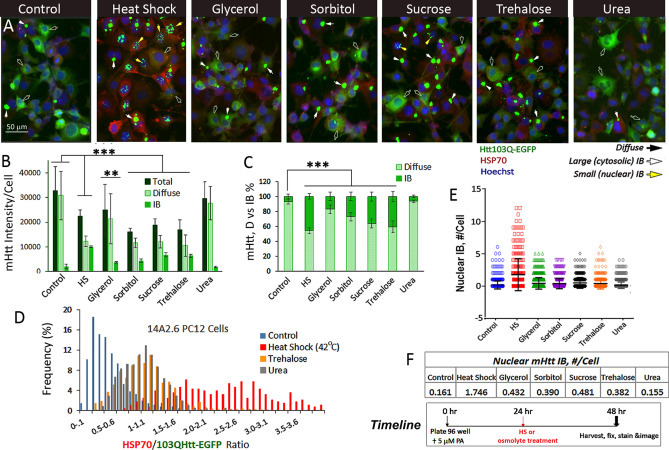
Osmolytes promote the compaction and aggregation of diffusible assemblies of 103Q mHtt-EGFP into forming inclusion bodies (IBs) in PC12 derived 14A2.6 neuronal cells. Cells were plated in 96 well plates. Ponasterone (PA) was added to a final concentration of 5 µM at plating (t = 0) to induce mHtt expression. Osmolytes (glycerol, sorbitol, sucrose, trehalose, and urea) were added at t = 24 h to a final concentration of 120 mM and continue to incubate at 37 °C for another 24 h. Heat shock of cells was done at t = 24 h by placing cells in a 42 °C incubator for 2 h and then returned to the 37 °C. Cells were harvested and fixed at 48 h and stained for HSP70 using a rabbit polyclonal antibody followed by Alexa Fluor 594 labeled goat anti-rabbit IgG secondary antibody. Nuclei were stained with Hoechst 33342. Cells were imaged with EVOS FL microscope and images were captured at the identical settings of exposure time and light intensity/contrast. (**A)** Representative image of the 103QHtt cells under different treatment conditions. Each frame shown represents an area of ~ 200 × 266 µm. Diffuse and cytoplasmic/nuclear IB forms of mHtt are represented, respectively, by black, white and yellow arrows and used throughout this work. The scale bar is of 50 µm. (**B)** Quantitation of mHtt signal intensity. Averaged intensities per unit cell of total, diffuse, and IB Htt-EGFP of images from four independent experiments with 20–40 captured images per condition. (**C)** Percentage of mHtt in diffuse versus IB format with the total being 100%. (**D)** Pivot chart profile of the HSP70/Diffuse mHtt (Red/Green) signal ratio of control, heat shock, trehalose- and urea-treated cells. (**E)** Nuclear IB counts. (**F)** Averaged nuclear IB count per unit cell. Timeline of experiment is indicated on bottom right of Figure. Full frame original images of Panel A are included in Supplementary Information section as Fig. S1A–G. The Macro program used for nuclear IB counting is included as Supplementary Method under SI. Probability of difference P > 0.05 is defined as not significant, between 0.01 and 0.05 is significant (*), < 0.01 is very significant (**), and < 0.001 is extremely significant (***).

We first asked the question of whether the physical state of mHtt in cells is changed when cells are treated with the stabilizing osmolytes: glycerol, sorbitol, sucrose and trehalose as well as the destabilizing osmolyte urea, all at 120 mM concentration (Fig. 1 and S1). In untreated control cells, mHtt was detected primarily as diffuse assemblies in the cytosol with an occasional cytoplasmic IB at 48 h after induction with 5 µM PA (Fig. 1A Control), and the IB:diffuse signal ratio was ~ 10%:90% (Fig. 1B,C). As a positive control, Fig. 1 shows that heat shock (42 °C for 2 h @ 24 h) spurred the compaction of diffuse mHtt into forming IBs to increase the mHtt IB signal to ~ 40–50% of total at the 48 h time point (Fig. 1A–C). The addition of stabilizing osmolytes at 24 h after PA induction similarly increased the compaction of diffuse mHtt into IB, with sucrose and trehalose being more potent (IB signal ~ 40% of total) than either glycerol or sorbitol (IB signal ~ 20% of total). Treatment with urea, a chaotrope^21,22^, had no significant effect on the total level of expression of mHtt or the IB:diffuse mHtt ratio and the mHtt profile was indistinguishable from that of the control (Fig. 1A–C). Heat shock and treatment with stabilizing osmolytes generally reduced the total expression of mHtt per unit cell (Fig. 1B); whether this is due to a decrease in mHtt synthesis or an increase in its clearance is not entirely clear^23^. We also evaluated the effects of methylamine osmolytes: trimethylamine N-oxide and N-methyl glycine (sarcosine) both promoted mHtt IB formation, but did so at cytotoxic concentrations and thus were not further studied.

While both heat shock and stabilizing osmolytes were effective in driving diffuse mHtt into IBs, their effects are distinguishable in two regards as follows. (1) Induction of HSP70: Whereas heat shock markedly increased the expression of HSP70, osmolytes had a more modest effect (Fig. 1A). Analysis of the HSP70/mHtt (red/diffuse green) signal ratio of individual cells showed distinct profiles for the control, heat shock, and osmolyte-treated cells (Fig. 1D). As expected, heat shock of cells resulted in a significant “red shift” in the HSP70/mHtt signal ratio, and the distribution curve showed a high kurtosis or tailedness indicative of the cell-to-cell heterogeneity in induction of HSP70 by heat shock (Fig. 1A). Cells treated with trehalose or urea had distribution curves of HSP70/mHtt signal ratio (red/diffuse green) in between that of the control and heat shock cells, showing a modest increase in HSP70/mHtt signal ratio over the control. The HSP70/mHtt distribution profiles of cells treated with glycerol, sorbitol or sucrose were similar to that of trehalose; a modest increase in HSP70 expression over the control was observed (data not shown). (2) Nuclear IB: Using a macro program to quantitate the number of nuclear IB (Supplementary Method), we observed that heat shock significantly increased the number of nuclear IB, entities distinguishable from the cytosolic IB in subcellular localization, size (nuclear < 1–2 µm vs cytoplasmic IB of 4–6 µm), and appearance (clustered vs. dispersed) (Fig. 1A,E). On the other hand, stabilizing osmolyte primarily spurred the formation of cytosolic IBs and had a much more limited effect in increasing nuclear IB formation (Fig. 1E,F). The destabilizing osmolyte urea had no effect on mHtt dynamics and the profile was similar to that of the control.

### Dose-dependent effects of stabilizing versus destabilizing osmolytes on mHtt remodeling

**Figure 2:**
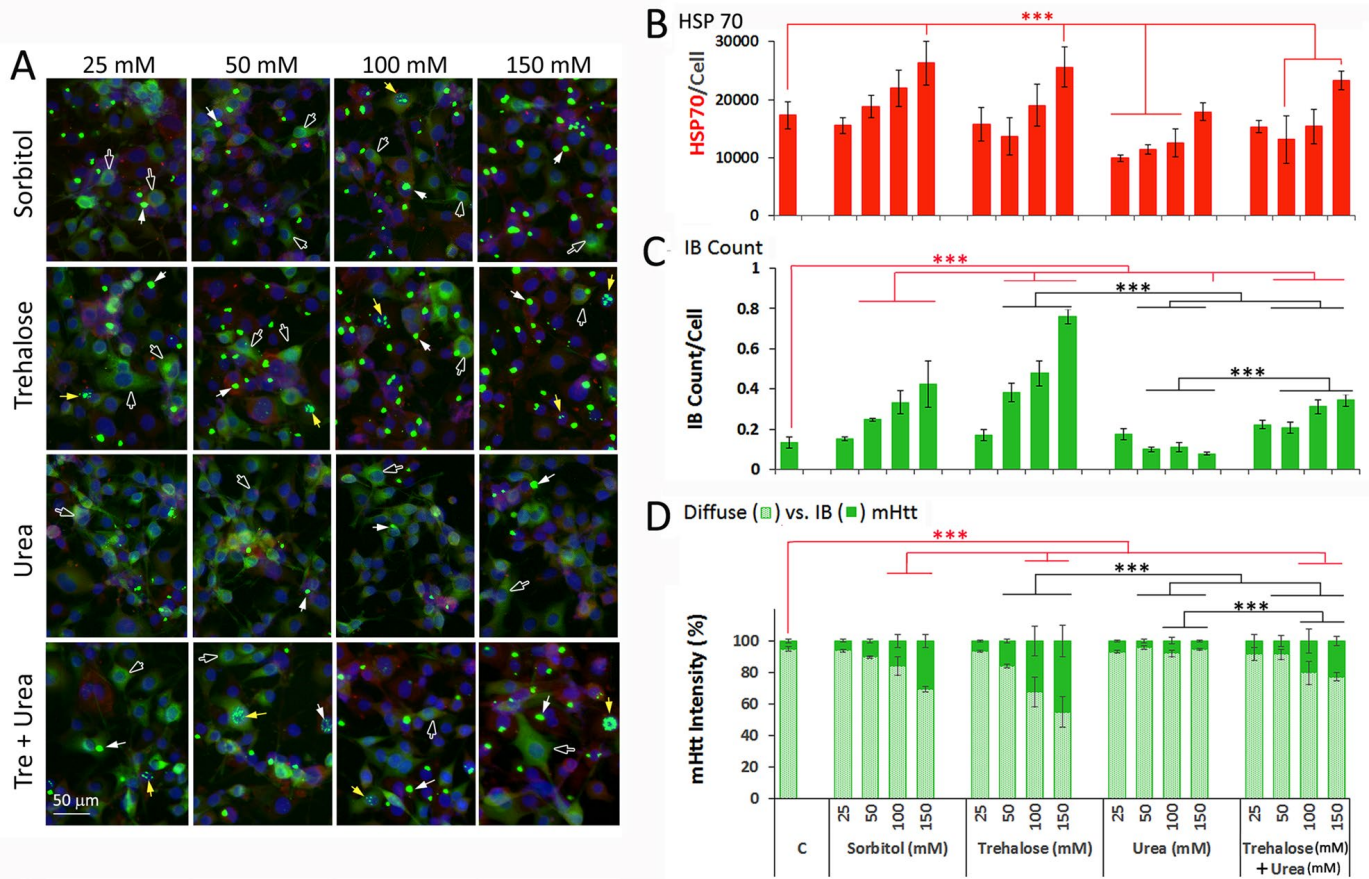
Osmolyte dose-dependent effects on HSP70 expression and distribution of mHtt in the diffusible ensemble (diffuse) versus inclusion body (IB) formats. The 14A2.6 line of PC12 cells were plated in 96 well plates. Ponasterone (PA) was added to a final concentration of 5 μM at plating (t = 0) to induce mHtt expression. Osmolytes (sorbitol, trehalose, urea, and trehalose + urea) were added at t = 24 h to final concentration of 25, 50, 100 and 150 mM as indicated and continue to incubate at 37 °C for another 24 h. The timeline of the experiment is as shown in Fig. 1. (**A)** Representative images of cells under the specified conditions. The scale bar is of 50 µm. (**B)** Quantitation of HSP70 of cells treated with specified concentrations of osmolytes. (**C)** Total IB count per unit cell. **D.** % distribution of mHtt signal in IB versus diffuse formats. Probability of difference P > 0.05 is defined as not significant, between 0.01 and 0.05 is significant (*), < 0.01 is very significant (**), and < 0.001 is extremely significant (***).

We then assessed the dose-dependent effect of osmolytes in induction of HSP70 vis-a-vis the remodeling of mHtt (Fig. 2). To ascertain the role of protein structuring in mHtt IB formation, we also tested the combined effects of a stabilizing and destabilizing osmolyte. Our standard protocol is to induce the expression of mHtt with 5 µM PA added at t = 0; osmolytes were added at t = 24 h to final concentrations as indicated, and cells were harvested, fixed and processed for HSP70 and nuclei staining at t = 48 h. (Note: The time-dependent effect of 100 mM trehalose on the % distribution of IB:diffuse mHtt is included in Fig. S2B.) Sorbitol and trehalose gave dose-dependent induction of HSP70 expression with the increase being highly significant (p < 0.001) at the 150 mM osmolyte concentration (Fig. 2A,B). Urea by itself had a limited effect on HSP70 expression and combining trehalose with urea was not significantly different from that of trehalose alone. Both sorbitol and trehalose significantly increased the compaction of diffuse mHtt into IB, with statistically significant increases in IB count and signal intensity at 50 mM of trehalose and 100 mM of sorbitol (Fig. 2C,D). Urea by itself reduced the mHtt IB count/cell at 150 mM as compared to the untreated control (Fig. 2C). When used in combination with trehalose, urea blunted the effect of equimolar concentration of trehalose by ~ 50% compared to trehalose alone in driving the assembly of diffuse mHtt into forming IB. Two other stabilizing osmolytes—glycerol and sucrose—also gave dose-dependent effects in promoting the compaction of diffuse mHtt into IB (Fig. S2A), an observation consistent with the innate and generic activity of osmolytes to drive protein structuring. In summary, our results show that stabilizing osmolytes, including glycerol, sorbitol, trehalose and sucrose, drive the assembly of diffuse mHtt into forming IB; that this effect is time- and dose-dependent and the formation of IB is blunted, but not abolished, by equi-molar concentrations of urea. We note that while urea is universally used as a “denaturing” agent at the 6–8 M concentration to scramble protein structure and function, at the mM concentration range used in this study, urea “relaxes” the tertiary structure of representative globular proteins without changing the native-like secondary structure and enzyme activity ^24^. Furthermore, we note that at concentrations < 150 mM, urea had no significant detrimental effect on cell morphology or viability for the duration of the experiment.

### Effect of osmolytes in promoting the aggregation of mHtt into IB is independent of HSC70 and HSP70 chaperones and distinct from inhibition of HSP90 or activation of autophagy

**Figure 3:**
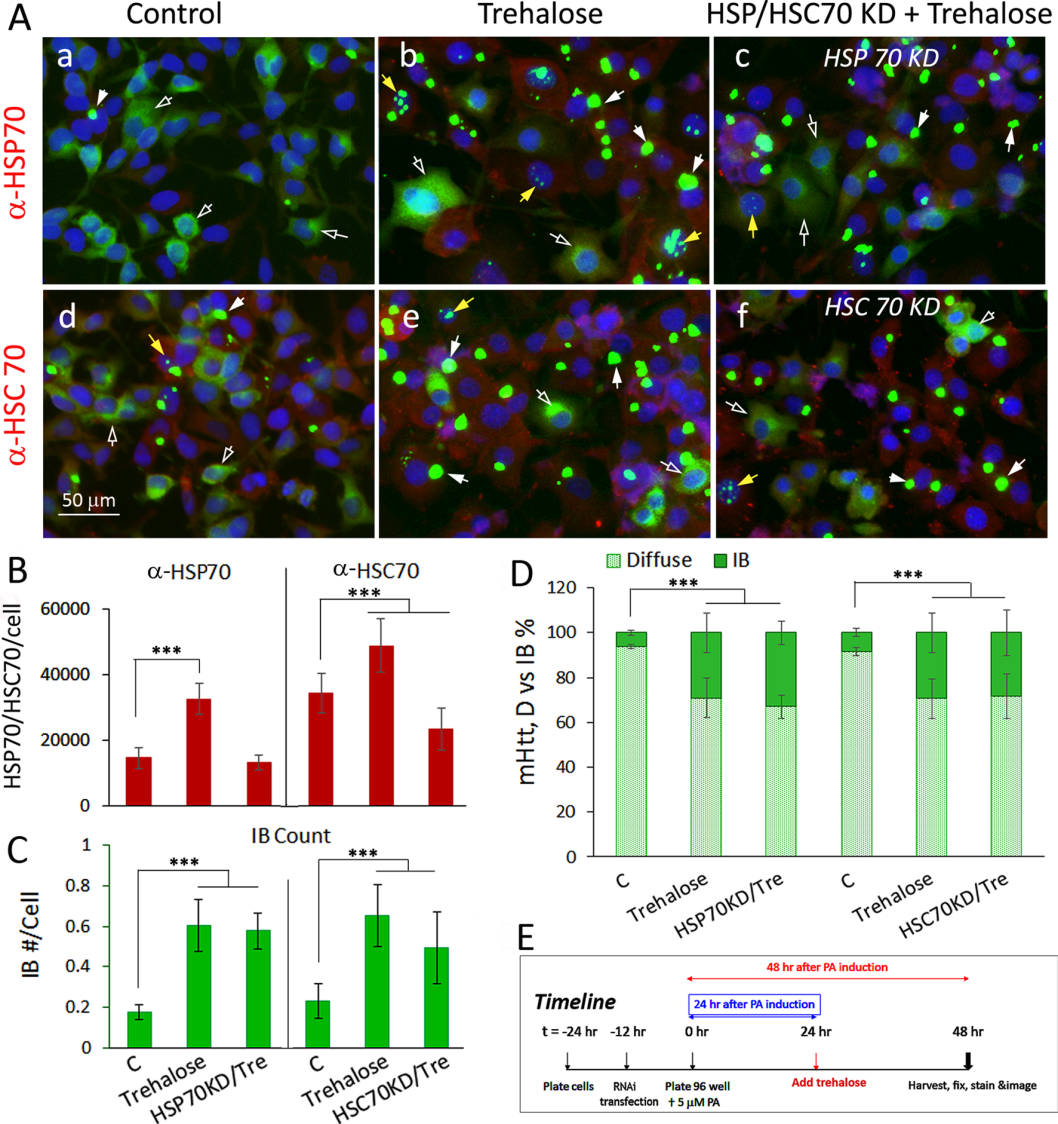
Effect of trehalose on the remodeling of mHttt into forming IB is unaffected by knocking down of HSP70 or HSC70 expression. For HSP70 KD, cells were transfected either with control or HSP70 DsiRNA according to methods previously described^9^. For HSC70 KD, cells were transfected with the hsc70 antisense vector DNA as described^9^. 12 h after transfection and designated as t = 0 in the timeline of the experiment, cells were plated in a 96 well plate in medium containing 5 µM PA to induce the expression of mHtt. Trehalose was added at 24 h after plating and cells were harvested, fixed and processed for imaging at 48 h. (**A)** Images of control and trehalose (120 mM) treated cells without and with HSP70 or HSPC70 KD. (**B)** Bar graph on HSP70 or HSPC70 expression of cells under the specified conditions. (**C)** Quantitation of the total IB count/cell. (**D)** % distribution of mHtt in the IB versus diffuse format. (**E)** Timeline of experiment. Result shown is the average of results from three separate experiments with 15–20 images per condition. Statistical analysis was done using GraphPad Instat. Probability of difference P > 0.05 is defined as not significant, between 0.01 and 0.05 is significant (*), < 0.01 is very significant (**), and < 0.001 is extremely significant (***).

HSP chaperones promote the folding/refolding and disposition of cellular proteins for maintenance of the proteome and recovery after stress^11,12^. Given that the heat shock mediated induction of HSP chaperones drives the compaction and assembly of mHtt into IB^9^ and our present observation that osmolytes modestly induced the expression of HSP 70 (Fig. 1, 2), we asked whether increases in HSP70 or HSC70 expression may contribute to the efficacy of osmolytes in driving diffuse mHtt into IB. For this, we tested the consequence of RNAi-mediated knockdown of HSP70 and HSC70 expression^9^ on trehalose-induced mHtt aggregation. Cell imaging and quantitation of HSP70 show that the trehalose induced increase in HSP70 expression was blocked by HSP70 RNAi knockdown (Fig. 3A,B). Trehalose also increased HSC70 expression and this was blocked by HSC70 antisense knockdown (Fig. 3A,B). Quantitation of mHtt IB count/cell (Fig. 3C) and of % of IB:diffuse mHtt signal ratio (Fig. 3D) showed that knocking down either HSP70 or HSC70 had no significant effect on the ability of trehalose to compact diffuse mHtt into IB. The expression and knockdown of HSP and HSC70 was confirmed by Western blot analysis (Fig. S3). The timeline of the experiment is as shown in Fig. 3E. These results show that trehalose has an innate activity, independent of HSP70 and HSC70 chaperones, in driving the compaction and aggregation of diffuse mHtt into IB.

Trehalose is neuroprotective and previous studies showed that trehalose, but not sucrose, raffinose or sorbitol enhances the clearance of mHtt and α-synuclein through autophagy^23^. Further, like many other metastable and aggregation prone regulatory proteins, mHtt and α-synuclein bind to and are clients of the HSP90 chaperone protein and inhibition of HSP90 increases the release and degradation of client proteins^25,26^. We show that treatment of cells with either AUY922 (40 nM) or rapamycin (0.2 µM) had little effect in promoting the compaction and assembly of diffuse mHtt into IBs as measured by IB signal/cell, and by IB count/cell (Fig. S4). Inhibition of HSP90 by AUY922 primarily depleted diffuse mHtt and this coincided with an increase in HSP70 expression. This result is consistent with previous observations that inhibition of HSP90 promotes the degradation of mHtt on one hand^25^ and trimerization and activation of HSF1 on the other^27^. Furthermore, we found that rapamycin (0.2 μM), an autophagy inducer, had no significant effect on the total mHtt expression, the % distribution of diffuse vs. IB forms of mHtt, or HSP70 expression within the dosage and time frame tested in our experiments.

### Stabilizing osmolytes promote mHtt IB formation and support survival under stress in conditionally immortalized striatal cells

To assess the generality of the effect of osmolytes in promoting the structuring and aggregation of polyQ-Htt, we sought to affirm key elements of our finding using a different cell model. The ST14A striatal cell line is derived from the striatum of the E15 rat embryo immortalized with a temperature sensitive large T antigen with a permissive growth temperature of 33 °C and restrictive temperature of 39 °C^28^. Previous studies clearly show that expression of the polyQ-expanded (120Q) but not the normal (15Q) Htt protein causes apoptotic cell death when challenged at the restrictive temperature by serum deprivation, with cell death occurring over time (48–120 h) in a highly reproducible manner^28^. Thus unlike the PC12 derived 14A2.6 cells, the ST14A cells are conditionally mortal and the consequence of disease protein expression as well as treatment conditions on cell survival under stress can be studied and determined.

**Figure 4:**
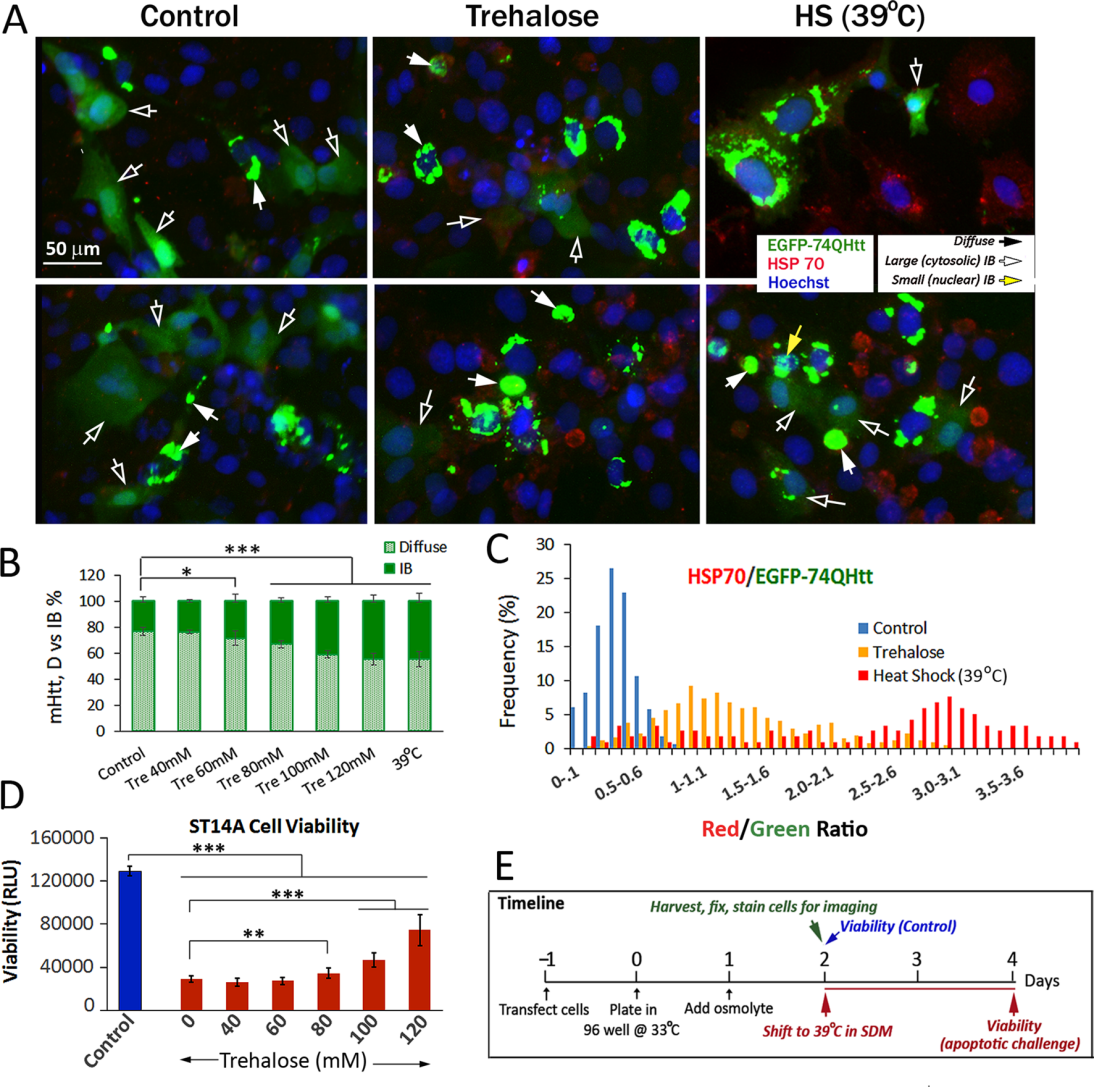
Trehalose and heat shock at 39 °C promoted the compaction of EGFP-Q74Htt in the ST14A striatal cells and supported cell survival under stress. ST14A cells were transfected with the pEGFP-Q74Htt plasmid DNA and plated in a 96 well plate (Day 0) according to methods described in the text. At t = 24 h, trehalose was added to a final concentration of 120 mM. For conditioning heat shock, cells were moved to 39 °C without serum deprivation. **(A)** Representative images (2 each) of control, trehalose-treated and 39 °C heat shock cells were fixed at t = 48 h and processed to stain for HSP 70 and nuclei according to methods described in the text. **(B)** Trehalose dose-dependent effect on the % distribution of mHtt signal in IB versus diffuse formats. Result from 39 °C heat shock cells is included for comparison. **(C)** Pivot chart of the HSP70/Diffuse mHtt (Red/Green) signal ratio of cells from control, 120 mM trehalose-treated and 39 °C heat shock samples. **(D)** ST14A cell viability. To relate osmolyte-induced changes in the material states of mHtt shown in panels A and B with the ability of the cells to withstand apoptotic insults, cells pre-treated with trehalose (0–120 mM) were shifted to the restrictive temperature of 39 °C in serum-starved medium (SDM)^28^. Cell viability was determined 48 h after initiation of this challenge and compared to that of the control at the start of this challenge. Probability of difference P > 0.05 is defined as not significant, between 0.01 and 0.05 is significant (*), < 0.01 is very significant (**), and < 0.001 is extremely significant (***). (**E)** Timeline of experiment.

ST14A striatal cells were transfected with pEGFP-Q74 Htt DNA (Addgene 40262). The effects of osmolytes and conditioning heat shock on mHtt compaction and aggregation of cells maintained in normal growth medium at the permissive temperature of 33 °C were determined. Both trehalose and heat shock at 39 °C promoted the compaction of diffuse EGFP-Q74Htt protein into IBs (Fig. 4A), with the effect of trehalose being dose-dependent (Fig. 4B). Trehalose also had a modest effect in increasing HSP70 expression (Fig. 4C), a result similar to observations made with the inducible PC12 cell model (Figs. 1, 2). To relate trehalose-induced changes in the material states of mHtt (diffuse vs IB) with the ability of the cells to withstand apoptotic insults, cells pre-treated with trehalose (0–120 mM) were shifted to the restrictive temperature of 39 °C in a serum-deprived medium (SDM) as previously described^28^. Cell viability was determined 48 h after initiation of this challenge and compared to that of the control at the start of this challenge. We observed a ~ 75% reduction in viability of the untreated cells 48 h after initiation of the apoptotic insult (Fig. 4D). Pre-treatment of cells with trehalose prior to the apoptotic challenge conferred a dose-dependent and statistically significant protection against death (Fig. 4D). A conditioning heat shock at 39 °C prior to the apoptotic challenge similarly conferred protection (data not shown). The timeline of the experiment is as shown in Fig. 4E. In sum, we show in ST14A striatal cells that trehalose promotes the compaction of diffuse mHtt into IB. This effect of trehalose is dose dependent and correlates with increased survival of cells subject to apoptotic stress.

### Effects of stabilizing and destabilizing osmolytes in rectifying CREB dysfunction

Dysregulation of transcription is an important pathogenic mechanism in HD and major changes in gene expression are reported in postmortem HD brains and in various experimental model systems^10,29,30^. In a previous study, we demonstrated that increased expression of diffuse mHtt quenches key transcription factor function implicated in stress resistance and memory formation including CREB, HSF1 and NFκB, while heat shock promotes aggregation of diffuse mHtt into IB and alleviates TF dysfunction^9^.

**Figure 5:**
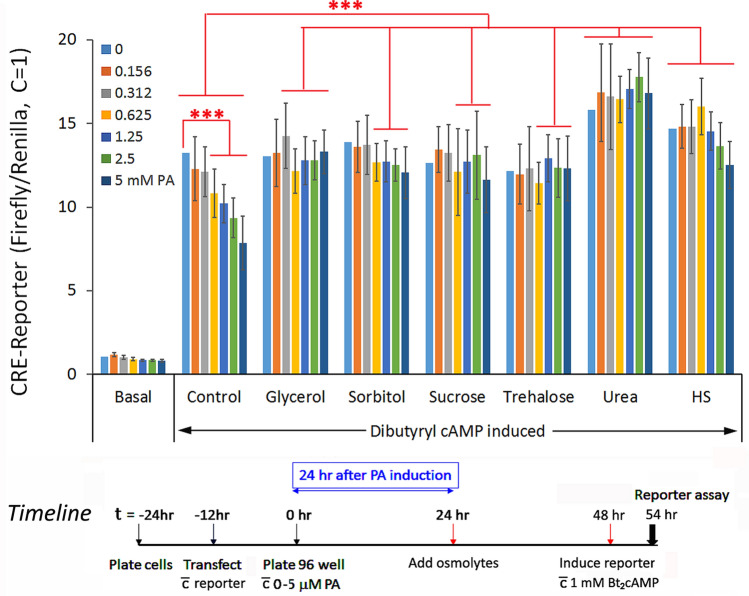
A comparison of the effects of stabilizing and destabilizing osmolytes versus that of heat shock in alleviating the diffuse mHtt dose-dependent repression of CRE-firefly luciferase reporter gene. The 14A2.6 line of PC12 cells were transfected with the CRE-firefly luciferase reporter DNA along with the internal control CMV-driven humanized Renilla luciferase reporter DNA as described^9^. After transfection, cells were plated at t = 0 into a 96 well plate and ponasterone (0–5 µM) was added to designated wells to final concentrations shown. Osmolytes (120 mM) were added at t = 24 h and incubation continued at 37 °C. Heat shock of cells was for 2 h at 42 °C at t = 24 h followed by recovery at 37 °C. The CRE-reporter gene was induced by the addition of 1 mM dibutyryl cAMP at t = 48 h and incubation for an additional 6 h at 37 °C. Reporter gene activity was assayed using the Dual-Glo reporter gene assay kit from Promega Inc. Results on the firefly luciferase reporter gene activity was normalized against that of the Renilla luciferase relative to the ratio of 1 for the un-induced control (basal; without PA or dibutyryl cAMP induction). Result is the average from three separate experiment each with 8 independent determinations. Probability of difference P > 0.05 is defined as not significant, between 0.01 and 0.05 is significant (*), < 0.01 is very significant (**), and < 0.001 is extremely significant (***).

Here, we asked if osmolytes have similar qualitative effects as heat shock in alleviating diffuse mHtt-mediated sequestration of CREB, a transcription factor centrally involved in memory formation^31,32^. To test this, cells were transfected with the CRE-firefly luciferase reporter along with the internal control Renilla luciferase and then plated into 96 well plate. At t = 0, defined concentrations of PA (0–5 µM) were added to individual wells of cells in 96 well plates for a graded increase in mHtt expression. Osmolytes (120 mM) were then added at t = 24 h to drive the compaction of diffuse mHtt into IB. Functional readout of CREB was assessed at the 48 h time point by adding 1 mM dibutyryl cAMP and incubation for 6 h at 37 °C prior to harvesting of the cells and assaying for reporter gene activity. Stabilizing osmolytes, glycerol, sorbitol, sucrose, and trehalose, effectively alleviated the mHtt-dose dependent repression of CREB reporter gene activity similar to the effect of heat shock (Fig. 5). Surprisingly, urea, a destabilizing osmolyte, while having no effect in promoting the aggregation of diffuse mHtt into forming IBs (Fig. 1, 2), negated the repressive effect of diffuse mHtt on CREB function. The “rebound” in CREB-reporter gene activity appeared to be comparable, if not higher, for urea than the stabilizing osmolytes. A possible explanation is that in addition to the sequestration of CREB by diffuse mHtt, other unknown cellular proteins (IDPs/IDRs) may also contribute to the quenching of CREB. In summary, our results suggest that osmolytes can reverse CREB dysfunction in our HD cell model by two apparently distinct mechanisms: (1) by promoting the structuring of mHtt into forming IBs effectively reducing the interactive surface area and negating the sequestration of CREB by diffuse mHtt as demonstrated with the stabilizing osmolytes, and (2) by disrupting the binding and sequestration of CREB by diffuse mHtt and perhaps other yet-to-be identified cellular proteins as shown by the effect of urea in de-repressing CREB activity without driving diffuse mHtt into forming IB.

## Discussion

In this work, we show that stabilizing osmolytes including glycerol, sorbitol, sucrose and trehalose share the generic activity of promoting the compaction and aggregation of mHtt from a diffuse ensemble into inclusion bodies (IBs). The minimal concentration of osmolyte needed to produce a statistically significant effect is ~ 50 mM with an optimal effect at the ~ 100–120 mM range after 24 h of treatment. Beyond ~ 150 mM, osmolytes have adverse effects on the growth, viability, and morphology of our cell systems, and this cytotoxicity is independent of mHtt expression. The effect of stabilizing osmolytes is innate, countered by the chaotrope urea, and unaffected by HSP70 and HSC70 knockdown. These results, along with our previous work of the effect of heat shock and HSP chaperone^9^, show that conditions that promote protein structuring drive the compaction and aggregation of diffuse mHtt into IBs. This change in the physical state of mHtt from diffuse to IB is correlated with alleviation of CREB dysfunction and enhanced cell survival under stress, results supporting the hypothesis that lower molecular weight entities of polyQ-expanded Htt are relevant pathogenic species in HD.

Carbohydrate osmolytes are neutral and biocompatible, do not interact with macromolecules in detrimental ways, and can be safely up- and down-regulated with minimal impact on overall cellular functions^14–17^. The concentration of osmolytes that cells accumulate under stress can be extraordinary and as high as 500 mM of trehalose in yeast^33^. The degree of protection conferred is profound as indicated by a > 20 °C increase in the thermal denaturation transition temperature of representative proteins^34^. In these and many other examples, osmolytes, through their direct effects on protein surface and peptide backbone and indirect effect on solvent water, change the folding landscape and shift the equilibrium towards the folded state^14–17^. As discuss below, our observation of the effect of osmolytes in driving the compaction and aggregation of diffuse mHtt protein to IB is consistent with this “structuring” theme of osmolytes. It extends the “pro-structuring” activity of osmolytes to include the class of “intrinsically disordered proteins” (IDPs)^35^, polyQ-expanded Htt as an example.

While many proteins adopt a well-defined structure for function, a large percentage of the proteome does not have a defined three-dimensional structure in the native and functional state^35–39^. Indeed, our proteome occupies the entire continuum from fully structured to completely disordered with the polyQ-expanded mHtt^exon1^ on the disordered end of this continuum. The “disordered” nature is due to the peculiarities of their amino acid composition—low in hydrophobic and high in charged amino acid residues—and is thus devoid of the necessary driving force to fold into a compact three dimensional structure. The distinct amino acid composition of IDPs results in their inability to form crystals. They have a large surface area and hydrodynamic dimension, a high proteolytic sensitivity, and a propensity to undergo liquid phase separation and condensation^40^. These properties are the basis of the development and use of computational tools for identifying and predicting such proteins^41^. The malleable and dynamic nature of IDPs in binding to different partners and their larger interacting surface for functionality per unit peptide length as compared to folded structures likely contributed to their selection and prevalence. Their importance is underscored by their disproportionate representation in transcription networks, chromatin organization, signaling pathways, phase-separated organelles, and neurodegeneration^36–39^.

A notable feature of IDPs is that the native or lowest Gibbs free energy state is composed of an ensemble of metastable entities with minimal differences in their free energy. Structuring of IDPs is entropy-dominated, and the energy landscape is inverted with respect to natively folded proteins^42,43^. Experimental analysis and simulation of the energy landscape of β-amyloid showed that the native and global free energy minimum consists of highly disordered structures, and higher free energy regions correspond to a library of metastable conformers with secondary structure elements that can more easily access aggregation pathways^42,43^. The inverted energy landscape contributes to difference in outcome of osmolyte-induced structuring in ordered versus disordered proteins, stabilizing the native fold to prevent denaturation and aggregation of ordered proteins versus formation of partially structured and aggregation prone metastable intermediates of IDPs.

The suggestion that mHtt structuring drives aggregation is consistent with the accepted idea of coupled folding and binding of IDPs^35,38,44^. Furthermore, there is clear evidence of the relatedness of Htt polyQ expansion, structuring, and aggregation. First, CD, EPR and NMR spectroscopy showed that with each Q residue there is an enhance secondary structure stability by 0.03–0.05 kcal/mol^45^. Second, molecular dynamics simulations showed that the average compactness and magnitude of conformational fluctuations increase with poly-Q length^46^. Third, in vitro aggregation kinetics analysis of polyQ peptides showed that the rate-limiting event in nucleated polymerization involves folding within the monomer^47^. Fourth, computation analysis of aggregation canonical free energy profiles showed a favorable downhill landscape for Q40 Htt compared to an uphill profile for Q20-30 protein^48^. Lastly, structural and functional analysis showed that, of the many different low molecular conformers of mHtt, a compact two-stranded hairpin conformer is most predictive of neuron vulnerability and HD pathogenesis^6,49^. Studies on other disease proteins provided similar insights. Thus, conditions that promote the structuring of α-synuclein enhance fibril formation ^50^. Likewise, pathogenic mutations of IκB (inhibitor of NFκB) and prion cause disorder-to-order transition^51,52^.

Collectively, these and related observations of a correlation between disease protein pathogenicity and the propensity to structure and aggregate led to a widely accepted proposition: that the aggregation of disease protein drives pathogenicity. We suggest an alternative hypothesis that can account for the pathogenic protein conformer driven pathogenesis: that the enhanced structuring propensity of disease proteins that drives their aggregation also spurs their binding to and quenching of regulatory protein factors to seed pathogenic cascades. The dynamics of homotypic aggregation versus heterotypic quenching would likely depend on the level of protein expression and accumulation, rates of IDP conformer conversion, and complementarity and binding rates between interacting elements^35^. Results presented in this work provide support for the two key elements of this hypothesis: that structuring drives disease protein aggregation, and inappropriate protein–protein interaction drives disease mechanism.

While much of the focus of this work is on the stabilizing osmolytes, observations made using the chaotrope urea ^13,21,22^ are instructive. In a previous study, stabilizing osmolytes have been shown to promote whereas urea halts the aggregation of the intrinsically disordered Tau protein aggregation^53^. It is also of interest and relevance that there is an increase in brain urea in the early prodromal phase in a HD animal model and in post mortem human HD brain tissues^54^. Our best estimate of this urea concentration is in the 40–50 mM concentration range. However, it is not known whether this increase in urea represents a pathogenic event as suggested^54^, or alternatively an innate compensatory mechanism to limit and rectify the pathogenicity of mHtt.

Differences in the effects of HS and osmolytes, the HS-induced increase in nuclear IB as an example, is likely attributable to differences in their mode of action in promoting protein structuring. The effect of protein chaperones may be motif-specific (e.g. hydrophobic patches), targeted, and can work in tandem (e.g. HSP70 and 40). The effects of osmolytes appear to be more chemistry driven, “polymer-directed,” and applicable to most, if not all, proteins by both direct and indirect action on the peptide backbone and water structure^15,16^. Polyol osmolytes do increase HSP/HSC70 expression; however, our results suggest that the effect of osmolytes on mHtt structuring and IB formation is independent of changes in HSP/HSC70 expression. Furthermore, there does not appear to be a qualitative difference in effects of the different stabilizing polyol osmolytes, including glycerol (a trihydric alcohol), sorbitol (a poly-alcohol), and disaccharides, such as trehalose and sucrose. In general terms, the disaccharides are more effective in promoting IB formation than the monosaccharide on a molar but not on a weight basis.

Importantly, while the compaction of mHtt^Exon1^ can negate some of the direct consequences caused by the binding of diffuse mHtt to regulatory protein such as CREB, the protein aggregates that form are not inert or innocuous. There are many examples in the literature that the deposition of both ordered (e.g. transthyretin, TTR) and disordered (e.g. β-amyloid) protein aggregates contribute to neuropathy, nephropathy, and cardiomyopathy^55,56^. In the case of HD, it has been shown that, whereas soluble mHtt causes apoptotic cell death, the formation of IB can trigger cellular quiescence, deactivate apoptosis, and lead to delayed necrosis^57^.

The maintenance of a functional proteome—keeping the ordered proteins folded and functional, supporting the dynamic structure and function of IDPs and IDRs, and disposing of dysfunctional and pathogenic proteins—is absolutely essential for the well-being of cells and organisms. There is a large body of evidence that during aging, there is a decrease in the integrity of the proteome due to decreases in chaperone mediated folding^58,59^ and clearance of damaged proteins through ubiquitin–proteasome and autophagy-lysosome systems^60^. These age-related dysfunctions are likely to have a synergistic effect to inflate the pathogenicity and blunt the disposition of mHtt aggregates that contribute to the demise of the post-mitotic neuron. These considerations underscore the need of multidisciplinary efforts to delineate and elucidate the relationship of the material states of mHtt to its pathogenicity in order to better understand HD pathogenesis for rational therapeutics development in HD.

## Materials and methods

### Materials

Osmolytes (ACS or molecular biology grade) were purchased from either Sigma Chem. Co. Fisher Scientific and dissolved in serum free medium or distilled water to a stock concentration of 1–2 M and added to cells to final concentrations as indicated. The HSP90 inhibitor AUY922 was from Apex Biotechnology, LLC. Rapamycin was from Sigma Chem. Co. Rabbit polyclonal antibody against HSP70—the RTG76 antibody that we generated against a recombinant histidine-tagged human HSP70 protein or sourced from Enzo Life Sciences (ADI-SPA812)—was used at 1:200 dilution for probing of the heat-inducible HSP70^9^. The SPA-816 rabbit polyclonal antibody (Enzo Life Sciences ADI-SPA-816) was used at 1:40 dilution for probing of HSC70. All other biochemical and chemical reagents were of molecular biology or reagent grade. siRNAs of rat HSP70 (rn.Ri.Hspa1a13.1, 13.2, and 13.3) was from Integrated DNA Technologies Inc. (IDT), and Hsc70 antisense vector was from Dr. Janice S. Blum’s laboratory of Indiana University Sch. of Med as described previously^9^. The pCRE-firefly luciferase reporter DNA (E8471), humanized Renilla luciferase DNA (phRLSV40), and the Dual-Glo luciferase assay reagent (E2920) were from Promega Inc. Lipofectamine 2000 reagent used for DNA transfection was from Invitrogen Co. The ST14A rat E14 striatal neuron derived cell line was from Coriell.org (CH00066; CHDI-90000066). Plasmid expression vectors pEGFP-Q23Htt (Plasmid #40261) and pEGFP-Q74Htt (Plasmid #40262) were from Addgene.Org.

### Cell culture

This study uses the PC12 derived cell line with stably integrated DNA of the 103Q Htt^Exon1^-EGFP sequences under the control of the inducible VgRxR promoter^9^. Briefly, cells were cultured and maintained in Dulbecco’s modified Eagle’s medium (DMEM) supplemented with 5% fetal bovine serum, 5% horse serum, 50 µg/ml streptomycin, 50 U/ml of penicillin, 100 µg/ml G418 and 200 µg/ml zeocin as previously described^20^. For imaging experiments, cells were plated in 96 Stripwell plates at a density of ~ 1–2 × 10^4^ cells/well. For reporter gene assay, cells were plated at ~ 5–6 × 10^4^ cells/well. Ponasterone (PA, Invitrogen) was added to induce the expression of mHtt-EGFP and the induction was monitored by live-cell imaging using an EVOS Fl microscope. Unless indicated otherwise, all treatments (osmolytes or heat shock) were initiated at 24 h after PA induction, and cells were fixed at 48 h followed by immuno-staining for HSP70 and cell imaging.

Culturing of the ST14A rat E14 striatal neuron derived cell line was done according to instructions provided by the HD Community Biorepository via Coriell Institute for Medical Research in DMEM with 10% fetal bovine serum. Cells were cultured and maintained at the permissive temperature of 33 °C, and freshly plated cells were transfected with the pEGFP-Q23Htt (Plasmid #40261; Addgene) and pEGFP-Q74Htt (Plasmid #40262) DNA. Transfected cells were treated with osmolytes or subjected to conditioning heat shock as indicated. Cells were fixed 24 h after treatment and processed for HSP/HSC70 and nuclear staining and imaging. Condition to evaluate the effects of apoptotic challenge by serum deprivation and incubation at the 39 °C restrictive temperature was as previously described^28^.

### Antibody probing, image acquisition and quantification

At the end of an experiment, cells were fixed and processed for immuno-staining for HSP70, nuclear staining and imaging as previously described^9^. In a given experiment, all images were captured under identical conditions. We wrote Macro programs to automate the quantitation of Htt intensity and scoring of individual cells for mHtt and HSP70 signal as previously described^9^. The macro program for quantitating nuclear IB counts of Fig. 1E is included under Supplementary Information/Supplementary Method.

### RNAi knockdown of HSP70 and HSC70 and assessment of HSP70 and HSC70 expression

A rat-HSP70 RNAi kit, TriFECTaRNAi, was obtained from IDT to knockdown HSP70 expression as previously reported^9^. The hsc70 antisense vector was generated by inserting hsc70 cDNA in an inverted orientation into pcDNAZeo(−)^9^. Freshly plated 14A2.6 cells were transfected with the designated DNA- or oligoRNA-lipid mixture. After transfection, cells were plated in 96 well plate and mHtt was induced by the addition of 5 µM PA and this is designated as t = 0. Treatment was done at 24 h after plating and cells were fixed and processed for imaging and quantitation at 48 h as previously described^9^. Western blot detection of HSP70 and HSC70 was done according to published methods and as described in legend of Fig. S3^61^.

### Reporter gene and cell viability assays

The cAMP-Response Element promoter (CRE)-firefly luciferase reporter gene activity was assessed as previously described^9^. The CRE-luciferase reporter DNA was transfected along with the internal control of phRLSV40 (synthetic humanized Renilla luciferase DNA). 12 h after DNA transfection, cells were plated into 96 Stripwell plates at a density of ~ 5–6 × 10^4^ cells/well (Corning/Costar 9102). Osmolytes (120 mM) were added at t = 24 h to drive IB formation, and functionality of CREB was assessed at the 48 h time point by adding 1 mM dibutyryl cAMP and incubation for 6 h at 37 °C prior to harvesting of the cells and assay for reporter gene activity. Results are the average ± standard deviation of four separate determinations of each sample/condition. Within each experiment, the reporter gene signal was tight with a < 10% sample-to-sample variation.

The Dual-Glo luciferase assay reagent from Promega Inc. (E2920) was used to assay for first the firefly then the Renilla luciferase activity according to manufacturer’s instructions as previously described^9^. Result of the CRE-firefly luciferase activity (in relative luminescence units, RLU) was normalized against that of the Renilla luciferase (RLU), and to facilitate comparison across experiments for statistical analysis this ratio was set at 1 for the control (without dibutyryl cAMP induction). By normalizing the CRE-firefly luciferase activity against that of the Renilla luciferase internal control, we effectively negated experimental variables such as differences in cell number, as well as non-selective effects of the treatment conditions/reagents on CRE-reporter gene expression.

### Data and statistical analysis

Image J Fiji and Macro programs were used for image analysis^9^. Statistics were done using GraphPad InStat. Data shown are the mean ± SD. The significance of difference between groups of data was done using ANOVA followed by post hoc Tukey–Kramer multiple comparisons test. Probability of difference P > 0.05 is defined as not significant (ns), between 0.01 and 0.05 is significant (*), < 0.01 is very significant (**), and < 0.001 is extremely significant (***).

## Supplementary information

Supplementary Information.
