# Supplementary material for: Osmolytes dynamically regulate mutant Huntingtin aggregation and CREB function in Huntington’s disease cell models
Source: Sci Rep. 2020 Sep 23;10:15511. doi: 10.1038/s41598-020-72613-3 (PMC7511939; doi:10.1038/s41598-020-72613-3)
Supplement: Supplementary file 1 — Supplementary Information. [file 41598_2020_72613_MOESM1_ESM.pdf]

SUPPLEMENTARY INFORMATION

TITLE: Osmolytes dynamically regulate mutant huntingtin aggregation and CREB function in Huntington's disease cell models

Authors: Shreyaas Aravindan, Samantha Chen, Hannaan Choudhry, Celine Molfetta, Kuang Yu Chen and Alice Y. C. Liu

Supplementary Figures (Figures S1-S4)

Figure S1. Representative images of 14A2.6 cells to illustrate the distribution of diffuse and IB forms of the mHtt protein of cells under different treatment conditions. The PC12 derived 14A2.6 cell line with a stably integrated RXR response element driven Htt103Q-EGFP construct was cultured and maintained according to conditions described in the text. mHtt expression was induced by the addition of 5μM ponasterone (an insect steroid hormone analogue) at the time of plating. For heat shock, cells were moved to 42°C for 2 hr at the 24 hr time point of PA induction and then returned to 37°C incubation. Osmolytes were added at t=24 hr to a final concentration of 120 mM and incubated at 37°C for another 24 hr. All cells were harvested and fixed at t=48 hr. To assess HSP70 expression, cells were probed with an anti-HSP70 rabbit polyclonal antibody followed by Alexa Fluor 594 goat anti-rabbit IgG secondary antibody and nuclei were stained with Hoechst 33342.

Figure S1a. Control 14A2.6 PC12 (Htt103Q) cells. Field dimension is 1200X900 micron. Total area is approximately 1mm^2.

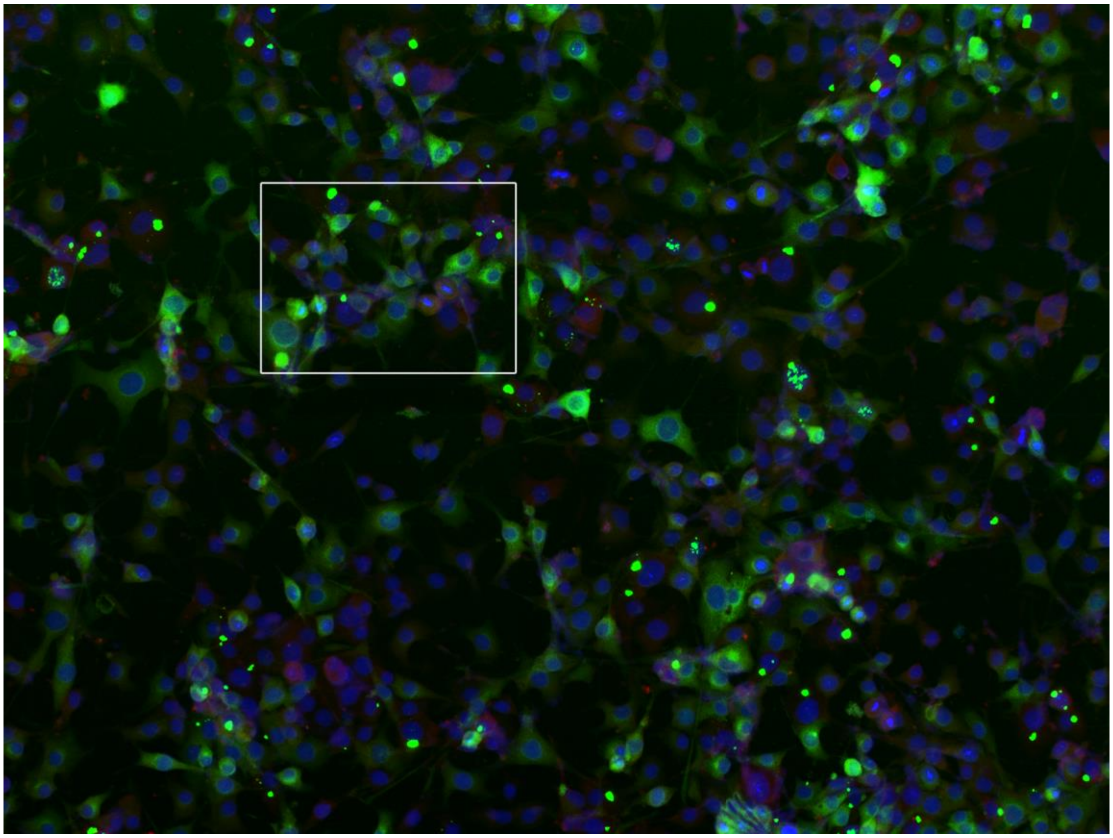

Figure S1B. Heat shock 14A2.6 PC12 (Htt103Q) cells. Field dimension is 1200X900 micron.

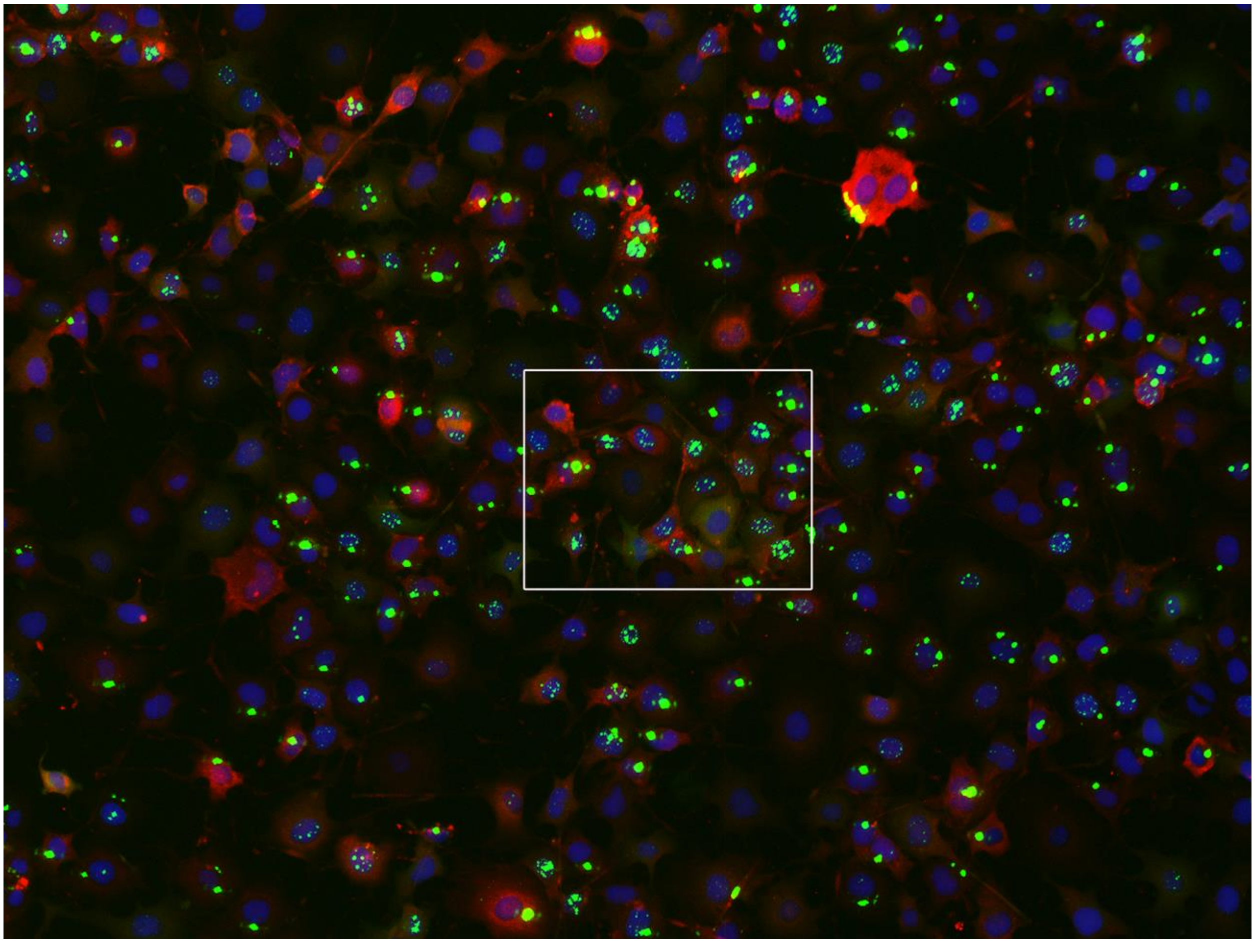

Figure S1C. 120 mM Glycerol treated 14A2.6 PC12 (Htt103Q) cells. Field dimension is 1200X900 micron.

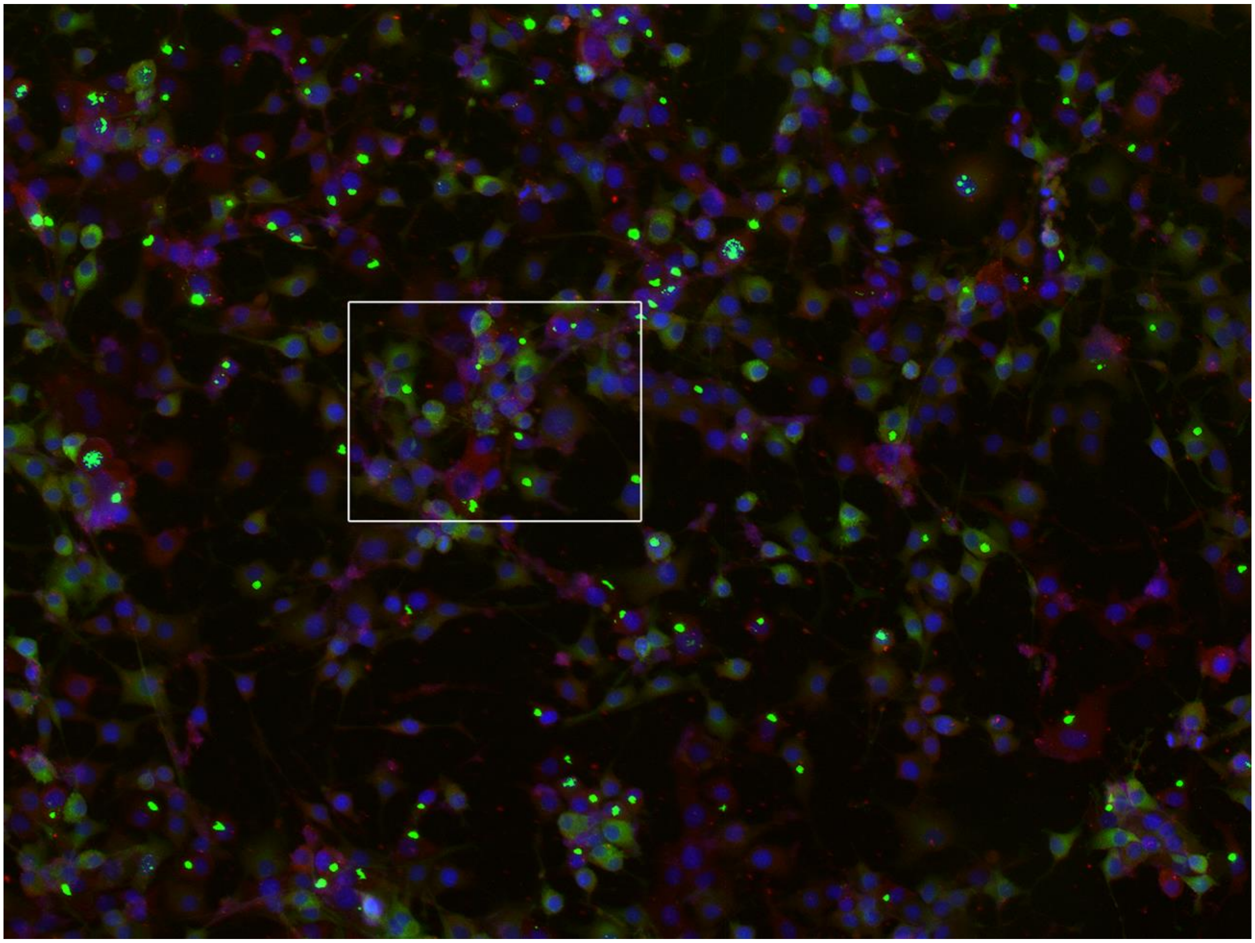

Figure S1D. 120 mM Sorbitol treated 14A2.6 PC12 (Htt103Q) cells. Field dimension is 1200X900 micron.

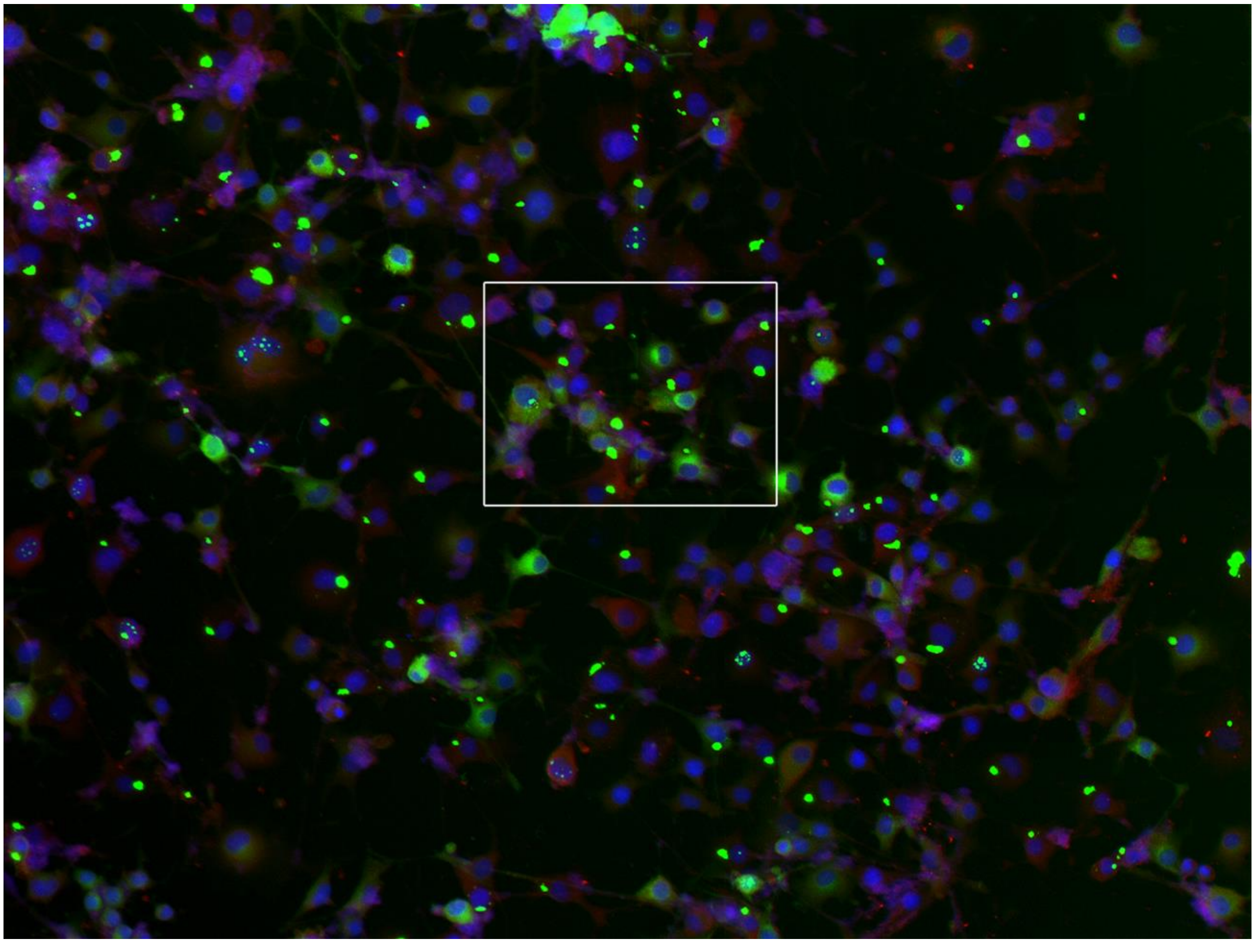

Figure S1E. 120 mM Sucrose treated 14A2.6 PC12 (Htt103Q) cells. Field dimension is 1200X900 micron.

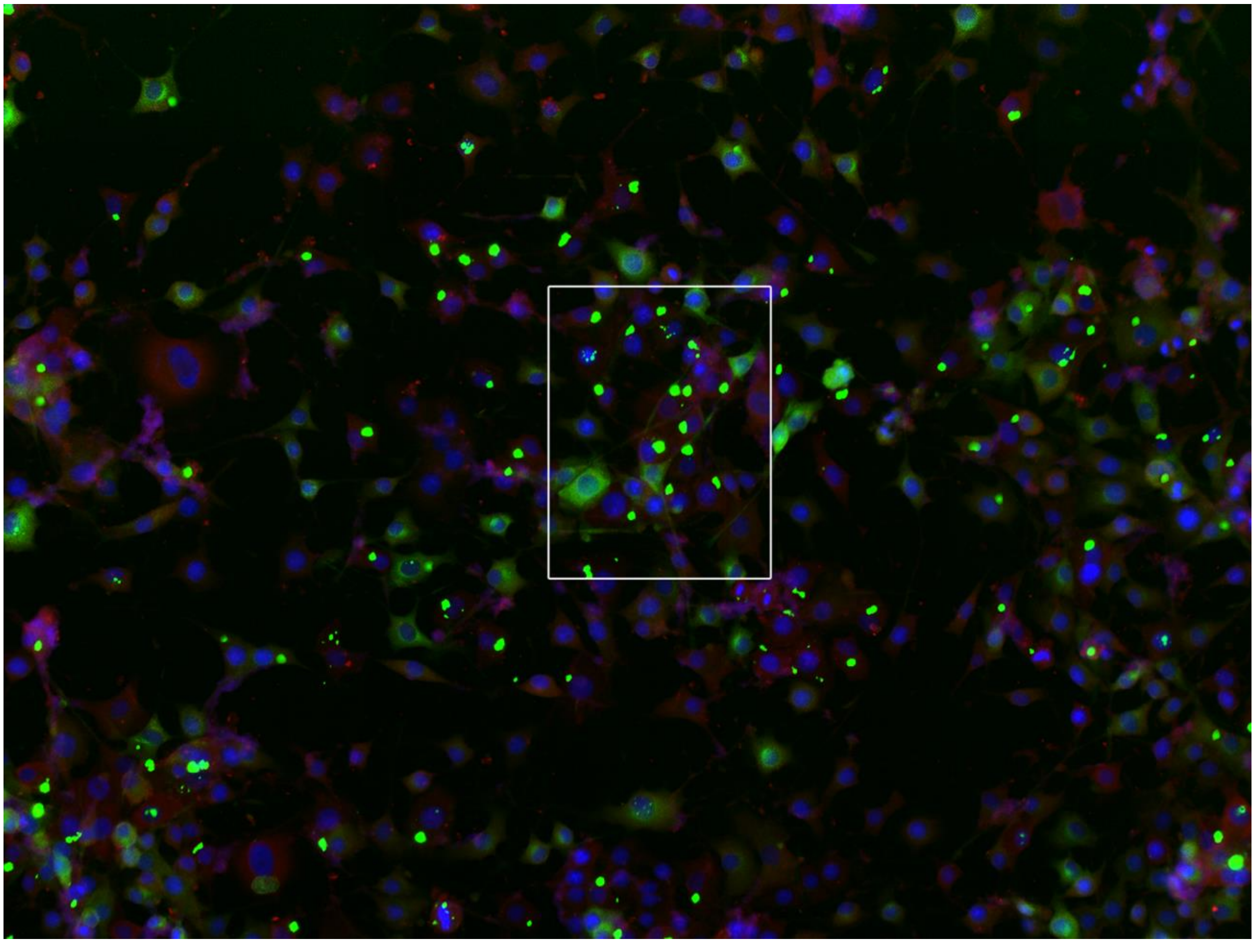

Figure S1F. 120 mM Trehalose treated 14A2.6 PC12 (Htt103Q) cells. Field dimension is 1200X900 micron.

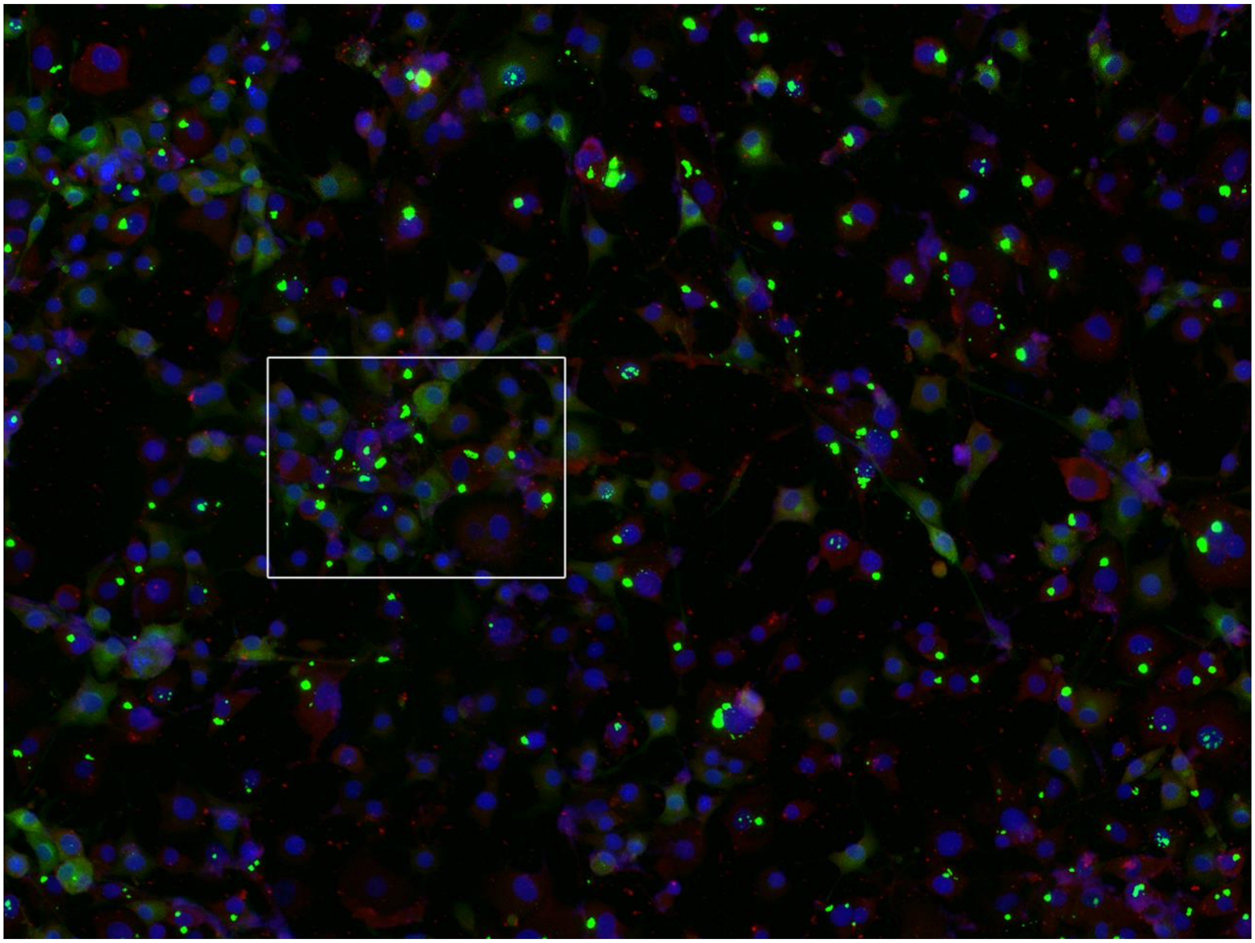

Figure S1G. 120 mM Urea treated 14A2.6 PC12 (Htt103Q) cells. Field dimension is 1200X900 micron.

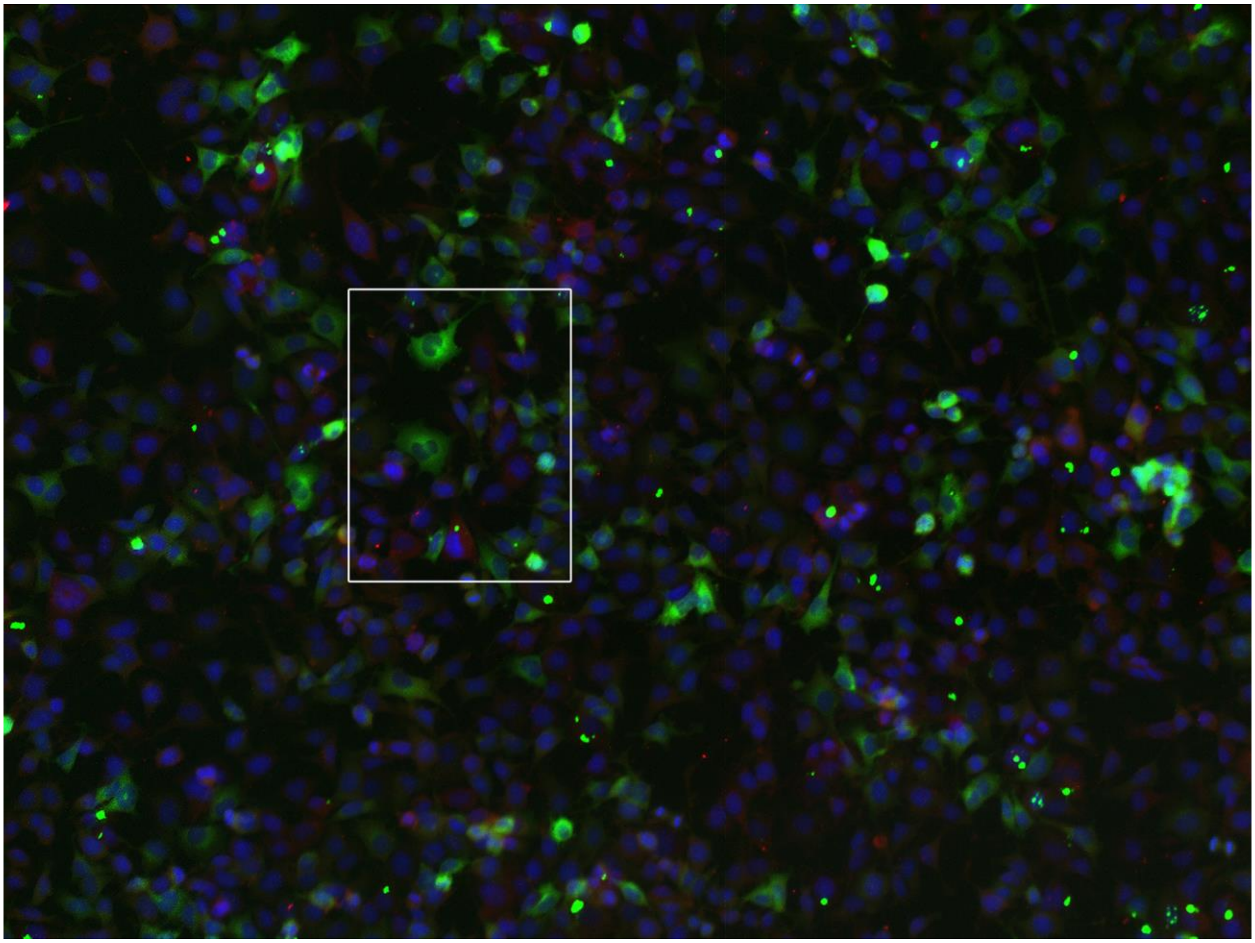

Figure S2. Dose response and time-dependent effects of osmolytes in driving the aggregation of diffuse mhtt into forming IB in 14A2.6 PC12 cells. (A) Osmolyte dose-dependent effects in remodeling diffuse mHtt into forming IBs. The timeline of experiment is as indicated in Methods. Briefly, cells were plated in 96 well plate with 5 μM PA to induce the expression of mHtt-EGFP. Osmolytes were added at t=24 hr to final concentrations as indicated and incubated at 37°C. Cells were harvested, fixed with 4% paraformaldehyde at 48hr and then processed for image analysis as described in the text. The bar graph presents the % of mHtt signal in diffuse and IB formats. **(B)** Time-dependent effect of 100 mM trehalose in the remodeling of diffuse mHtt into forming IBs. The details of the experiments were as described in Part A of this figure except cells were harvested at the indicated time points after the addition of 100 mM trehalose. Result represents the average of 15-20 images for each condition in dose and time. Statistical analysis was determined using Anova followed by post hoc Tukey-Kramer multiple comparisons test. Probability of difference P>0.05 is defined as not significant, between 0.01 and 0.05 is significant (*), <0.01 is very significant (**), and <0.001 is extremely significant (***).

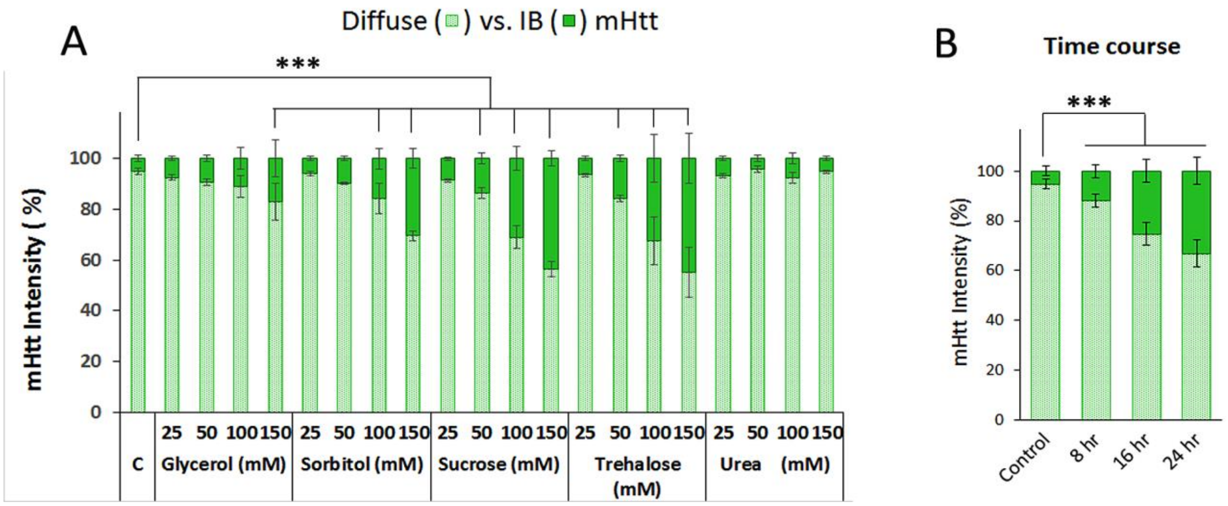

<u>Figure S3. Western blot analysis of HSP and HSC 70 knockdown.</u> Transfection was done as described for experiments shown in Figure 3. Mock- and vector-transfected cells were plated in 35 mm plates and maintained in culture for 24 hr. Cells were heat shock at 42°C for 2 hr followed by recovery at 37°C for 22 hr. Trehalose was added to a final concentration of 120 mM and cells incubated at 37°C for 24 hr. Cells were harvested and processed for immune-Western blot detection of HSP and HSC 70 according to methods previously described [61]. The positions of the 72 kDa HSP70 and the 73 kDa HSC70 protein are shown and their relative intensities are as indicated at the bottom of the figure.

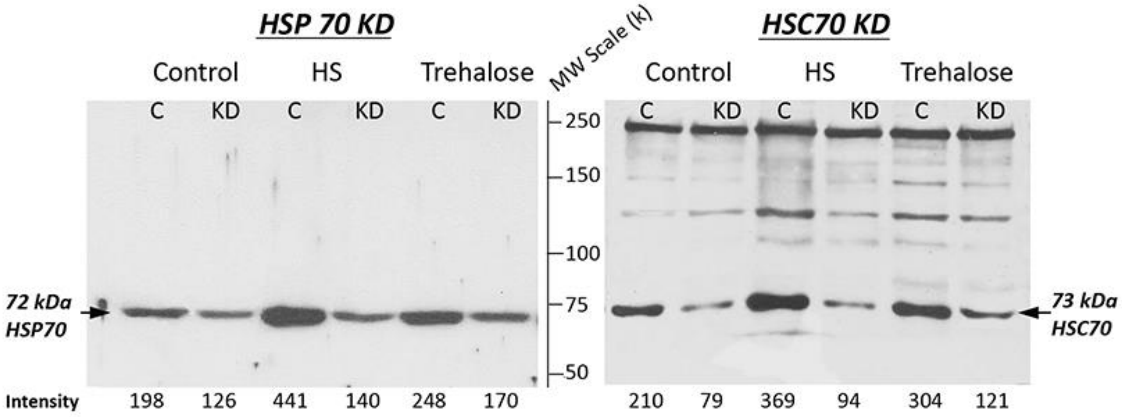

Figure S4: A comparison of the effects of AUY922, rapamycin, trehalose, and heat shock on mHtt dynamics. The expression of mHtt-EGFP was induced by the addition of 5 μM PA as described in the legend of Figure 1. The different treatments were applied at t=24 hr, with 40 nM AUY922 to inhibit HSP90 and 0.2 μM of rapamycin to induce autophagy. Heat shock was at 42°C for 2 hr followed by recovery at 37°C for 22 hr. All cells were harvested and fixed at 48hr and stained for HSP70 and nuclei. Each frame shown represents an area of ~200 X 266 μm. Quantitative results represent the average of 10-20 images from three separate experiment over a period of ~12 months. **A:** Representative images from control, 50 nM AUY922-, 0.2 μM rapamycin-, 100 mM trehalose-treated cells as well as HS cells (2 hr, 42°C @ 24 hr) are shown in Panels A: a-e, respectively. **B.** Quantitation of the total, diffuse and IB forms of mHtt under the different treatment conditions. **C.** % distribution of mHtt signal in the IB versus diffuse format. **D.** IB count per cell. Result is the average ± standard deviation of the mean. Probability of difference P>0.05 is defined as not significant, between 0.01 and 0.05 is significant (*), <0.01 is very significant (**), and <0.001 is extremely significant (***).

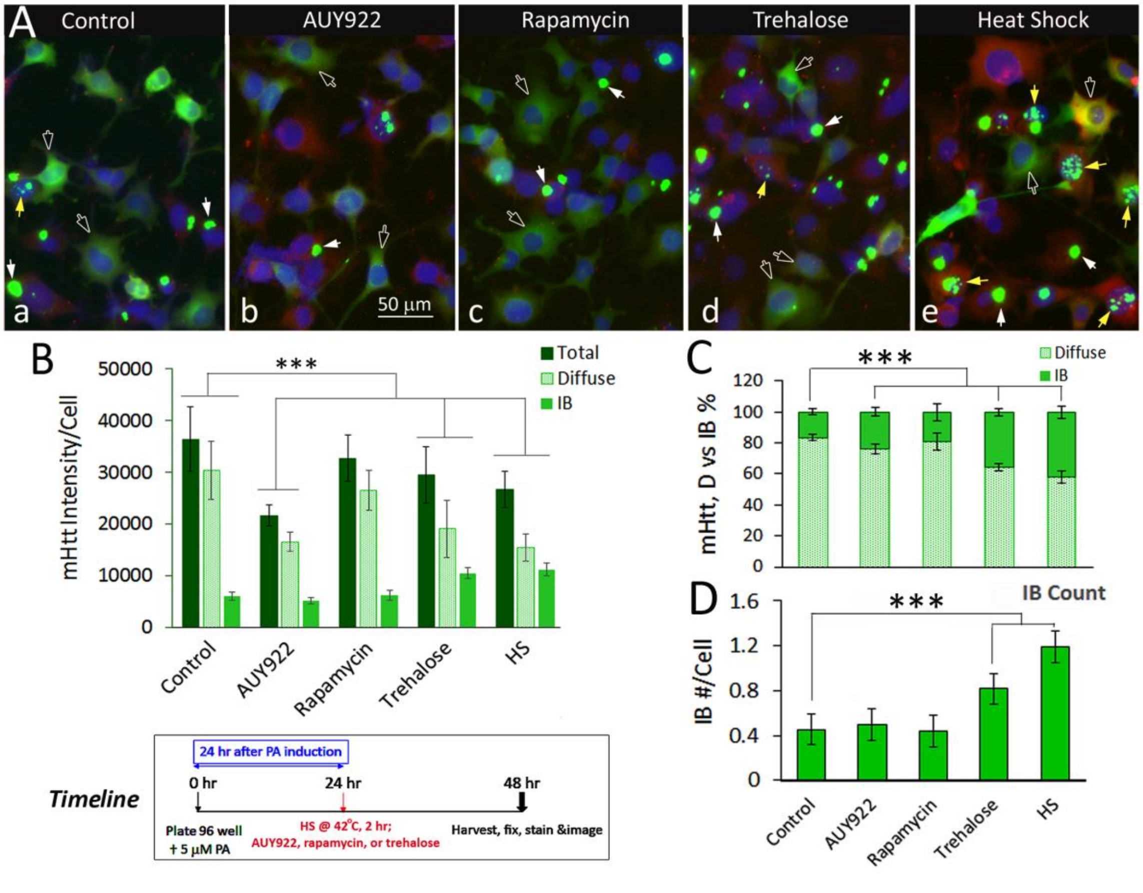

Supplementary Method: Macro program for nuclear IB count per cell.

```
//For use in Fiji (ImageJ)
//This macro will need to process images one by one due to the
//background in the blue images varying greatly from image to image
//This will measure the number of IB's in each nucleus.
//AL modified this such that Nuclear Size is 80-infinity (this is about 7.4 micron; changed 6/26/19 to 60 - 6.42 micron); Nuclear intensity 50-255; IB 130-255
//Delete "Dilate" from Justin's original; keep "Watershed"

//Size range of Nucleus = S -> T
S = "80";
T = "Infinity";

//Size range of IB = X -> Y
X = "0";
Y = "Infinity";

//Intensity range of the nucleus in blue channel = N -> 255
//It is advisable to be more stringent of your selection of the
//threshold to be more exclusive to the nucleus over the background,
//especially because we dilate the selection in the macro - this was removed 2019.
N = "50";

//Intensity range of the IB = Z -> 255
Z = "130";

dir = getDirectory("image");
name = getTitle;
output = getDirectory("Choose Output Directory");

run("Split Channels");
selectWindow(name+" (blue)");
run("Duplicate...", "title=Nucleus");
selectWindow(name+" (green)");
run("Duplicate...", "title=NuclearROI");

//ROI Selection
selectWindow("Nucleus");
setAutoThreshold("Default dark");
run("Threshold...");
setThreshold(N,255);
setOption("BlackBackground", false);
run("Convert to Mask");
run("Watershed");
run("Analyze Particles...", "size=S-T summarize add");

//Thresholding for IB
```

```
selectWindow("NuclearROI");
setAutoThreshold("Default dark");
run("Threshold...");
setThreshold(Z,255);
setOption("BlackBackground", false);
run("Convert to Mask");
run("Watershed");

//Counts the number of ROIs in the ROI manager and uses this to set up an end condition
//for the while loop which measures each ROI one by one in the manager.
selectWindow("NuclearROI")
end=roiManager("count");
a=1;
while(a<=end) {
	b=a-1;
	roiManager("Select", b);
	run("Analyze Particles...", "size=X-Y summarize");
	a=a+1;}
```
